# Vestibulospinal and Corticospinal Modulation of Lumbosacral Network Excitability in Human Subjects

**DOI:** 10.3389/fphys.2018.01746

**Published:** 2018-12-06

**Authors:** Dimitry G. Sayenko, Darryn A. Atkinson, Amber M. Mink, Katelyn M. Gurley, V. Reggie Edgerton, Susan J. Harkema, Yury P. Gerasimenko

**Affiliations:** ^1^Department of Integrative Biology and Physiology, University of California, Los Angeles, Los Angeles, CA, United States; ^2^Center for Neuroregeneration, Department of Neurosurgery, Houston Methodist Research Institute, Houston, TX, United States; ^3^Neuroscience Collaborative Center, Frazier Rehab Institute, Louisville, KY, United States; ^4^Department of Neurological Surgery, University of Louisville, Louisville, KY, United States; ^5^Department of Physiology and Biophysics, University of Louisville, Louisville, KY, United States; ^6^School of Medicine, Louisiana State University, New Orleans, LA, United States; ^7^Pavlov Institute of Physiology, St. Petersburg, Russia

**Keywords:** electrophysiological assessment, transcutaneous electrical spinal cord stimulation, galvanic vestibular stimulation, transcranial magnetic stimulation, neurorehabilitation, spinal cord injury

## Abstract

As part of a project aimed to develop a novel, non-invasive techniques for comprehensive assessment of supraspinal-spinal connectivity in humans, the present study sought to explore the convergence of descending vestibulospinal and corticospinal pathways onto lumbosacral motor pools. Transcutaneous electrical spinal stimulation-evoked motor potentials were recorded from knee and ankle flexors and extensors in resting neurologically intact participants. Descending influences on lumbosacral motor neurons were studied using galvanic vestibular (GVS) or transcranial magnetic stimulation (TMS) to elicit descending vestibulospinal or corticospinal volleys, respectively. Facilitatory conditioning effects of descending corticospinal volleys were manifested by a significant increase of spinally evoked motor potentials in recorded knee and ankle muscles bilaterally, and were observed at the 10–30 ms conditioning-test intervals (CTIs); whereas, facilitatory conditioning effects of vestibulospinal volleys manifested at longer latencies (CTIs of 90 and 110 ms), and lasted up to 250 ms. TMS mediated volleys revealed the conditioning effects at both short and long latencies, suggestive of both direct and indirect influence. In contrast, vestibulospinally mediated conditioning effects occurred at longer latencies, consistent with this pathway’s known anatomical and functional interfaces with other descending systems including the reticulospinal pathway and, suggestively, propriospinal interneurons. Our work demonstrates the utility and sensitivity of transcutaneous spinal stimulation in human neurophysiological studies as a technique for quantitative characterization of excitatory conditioning effects in multiple lumbosacral motor pools, obtained through descending pathways. This characterization becomes critical in understanding the neuroplasticity in the central nervous system during motor learning and neurological recovery.

## Introduction

Recent reports of the recovery of voluntary movement in individuals with complete chronic paralysis (Angeli et al., 2014, 2018; Gerasimenko et al., 2015; Hofstoetter et al., 2015b; Grahn et al., 2017; Gill et al., 2018) raise an obvious question as to the neural substrates mediating the descending connections between the brain and spinal cord below the lesion (Minassian and Hofstoetter, 2016; Taccola et al., 2018), and demonstrate the need for quantitative assessment techniques for evaluation of the existing and/or *de novo* functional connectivity between supraspinal and spinal networks. Such dynamic physiological phenomena cannot be assessed with conventional techniques. The present work focuses on quantitative characterization of the function of vestibulospinal and corticospinal tracts, critical for postural regulation and motor control (Horak and Nashner, 1986; Horak et al., 2001; Jacobs and Horak, 2007; Deliagina et al., 2012).

The conditioning effects of galvanic vestibular stimulation (GVS) (Iles and Pisini, 1992b; Kennedy and Inglis, 2001, 2002; Fitzpatrick and Day, 2004; Kennedy et al., 2004a; Ghanim et al., 2009; Lowrey and Bent, 2009) and transcranial magnetic stimulation (TMS) (Iles and Pisini, 1992a; Nielsen et al., 1993; Meunier, 1999; Poon et al., 2008; Costa et al., 2011; Guzman-Lopez et al., 2012; Leukel et al., 2012) on excitability of lower motor neurons, have been examined previously, principally using the H-rereflex. Although the H-reflex is limited in that it only assesses a single unilateral spinal motor pool, several guiding principles and observations can be derived from these studies. Importantly, GVS induced volleys produce long-lasting effects on the H-reflex excitability, with the most pronounced responses at condition-test intervals (CTIs) of approximately 80 to 140 ms (Kennedy et al., 2004b; Ghanim et al., 2009; Lowrey and Bent, 2009). Modulatory effects occurring at such long latencies likely reflects polysynaptic vestibulospinal transmission, suggestive of integration with other descending inputs. In fact, earlier studies indicate that GVS induced responses are not conveyed exclusively through the vestibulospinal pathways, and can be transmitted, at least partially, via reticulospinal tract (Pierrot-Deseilligny and Burke, 2005; Lowrey and Bent, 2009). However, the net effect of GVS induced volleys on multi-segmentally evoked lumbosacral motor responses in different proximal and distal lower limb muscles remains unknown.

The facilitating effects of conditioning TMS on the H-reflex were shown to be prominent first at short latency CTIs of up to 35 ms, and attributed to primarily monosynaptic descending corticospinal projections (Valls-Sole et al., 1994; Nielsen et al., 1995; Costa et al., 2011). Interestingly, a second, long-latency phase which peaks up at 70–100 ms was detected and attributed to an excitatory descending volley in subcortical motor tracts activated by TMS, a coincident segmental input from excitatory heteronymous Ia afferents generated in simultaneously activated muscles, or a startle-like reaction (Holmgren et al., 1990; Dimitrijevic et al., 1992; Nielsen et al., 1993; Goulart et al., 2000; Costa et al., 2011; Guzman-Lopez et al., 2012).

Most recently, the multisegmental effects of the corticospinal convergence were studied using a combination of TMS and transcutaneous, or transspinal, electrical spinal cord stimulation (TSS) (Knikou, 2014; Roy et al., 2014; Andrews et al., 2015). It was shown that corticospinal volleys can simultaneously facilitate spinally evoked potentials from multiple lumbosacral motor pools (i.e., in multiple knee and ankle muscles) with temporal manifestation similar to the H-reflex studies (with correction to the H-reflex latency). TSS is a convenient tool to modulate spinal motor excitability through activation of the corresponding posterior roots (Troni et al., 2011; Roy et al., 2012; Krenn et al., 2013; Sayenko et al., 2015a). Spinally evoked motor potentials are reminiscent of the monosynaptic H-rereflex (Courtine et al., 2007; Minassian et al., 2007a), but owing to the convergence of the sensory fibers at the lumbosacral enlargement, stimulation delivered over the posterior roots entering the lumbosacral enlargement can generate evoked potentials in multiple proximal and distal leg muscles bilaterally and simultaneously. Such resolution level in characterization of multiple motor pool excitability becomes particularly valuable in efforts to understand the reorganization strategies of the brain and spinal cord during motor learning and/or functional recovery after neurological disorders or lesions, such as spinal cord injury (SCI).

The present study was designed to systematically investigate and differentiate the multi-segmental convergence of two distinct descending supraspinal pathways on lumbosacral motor pool excitability in neurologically intact participants. We hypothesized that: (1) the temporal profiles of net spinal motor output modulation in response to GVS or TMS elicited descending volleys will allow distinction between the vestibulospinal and corticospinal routes of transmission, and (2) modulation of spinally evoked motor potentials in various leg muscles in response to a specific source of descending influence will depend on the rostro-caudal or ipsi-contralateral location of projecting motor pools, or on their functional role in motor control, e.g., flexors vs. extensors. To assess these hypotheses, we examined the effects of vestibular or cortically induced volleys on the amplitude of spinally evoked motor potentials in multiple leg muscles in neurologically intact human participants. We demonstrate that motor output of multiple spinal segments in humans can be modulated with different spatiotemporal manifestation, depending on the specific route of supraspinal-spinal transmission.

## Materials and Methods

### Participants

The experiments were conducted on 20 (7 males and 13 females; mean ± SD: age 29.5 ± 4.9 yrs, height 166.0 ± 9.2 cm, body mass 67.6 ± 11.6 kg) and 8 (4 males and 4 females; mean ± SD: age 29.3 ± 4.9 years, height 172.9 ± 10.6 cm, body mass 74.1 ± 12.9 kg) volunteers during GVS and TMS conditioning sessions, respectively. None of the participants had any history of neurological disorders. Written informed consent was obtained from all participants. The present study conformed to the standards set by the *Declaration of Helsinki*, and the procedures were approved by the University of Louisville (KY, United States) institutional review board.

### EMG Recording and Data Collection

Surface electromyogram (EMG) signals were recorded bilaterally using bipolar surface electrodes (Motion Lab Systems, Baton Rouge, LA, United States) placed longitudinally on the vastus lateralis (VL), rectus femoris (RF), and medial hamstring (MH), tibialis anterior (TA), soleus (SOL), and medial gastrocnemius (MG) muscles with fixed inter-electrode distance of 17 mm. Indifferent, or ground reference electrode was placed over the distal part of the left tibia bone, and connected to the 16-channel portable unit which carried the EMG signals to the desktop interface unit. The EMG signals were differentially amplified using MA300 EMG System (Motion Lab Systems, Baton Rouge, LA, United States) with a band-pass filter of 10 Hz to 2 kHz (-3 dB). The EMG data were digitized at a sampling rate of 5000 Hz.

### Transcutaneous Electrical Spinal Cord Stimulation

A custom-built constant current stimulator with a range of 0–100 mA was used to deliver transcutaneous spinal cord stimulation. Similar to the procedures described in previous studies (Courtine et al., 2007; Minassian et al., 2007b; Hofstoetter et al., 2008, 2018; Sayenko et al., 2015a), the stimulation was administered using a conductive rubber electrode with a diameter of 18 mm placed on the skin at one of the spaces between the spinous processes of the T10 and T11, T11 and T12, or T12 and L1 vertebrae at the midline over the vertebral column as a cathode, and two 5 × 9 cm self-adhesive electrodes (Pro-Patch, Taiwan) located symmetrically on the skin over the iliac crests as anodes. Stimulation location was selected such that activation thresholds of proximal muscles were observed at lower stimulation intensities relative to distal leg muscles. This ensured that stimulation was applied along the most rostral portion of the lumbosacral enlargement, in order to standardize the procedure across participants, as well as to ensure predominantly afferent fibers’ activation and to minimize the possibility of stimulation of mixed peripheral nerves from the most caudal segments as they descend outside the spinal cord (Minassian et al., 2007a; Troni et al., 2011; Roy et al., 2012; Sayenko et al., 2015a). The stimulation was delivered as single, 1 ms, monophasic, square-wave pulses, with inter-stimulus interval randomized between 6 and 10 s.

### Galvanic Vestibular Stimulation (GVS)

Bipolar binaural GVS was delivered via two electrodes (Ag/AgCl) placed on the skin over the participant’s right and left mastoid processes (Lowrey and Bent, 2009). The electrode on the right was used as a cathode, and the electrode on the left was used as an anode. The GVS conditioning pulse duration was 300-ms, at an intensity that did not produce uncomfortable sensations of pinching or sharp pricks behind the ears, yet caused a perception of head displacement (amplitudes ranged from 2 to 5 mA) (Kennedy and Inglis, 2001, 2002; Lowrey and Bent, 2009). The conditioning GVS preceded the test responses at different conditioning-test intervals (CTIs) which were measured as the time between the onset of the conditioning pulse and spinal stimulation. Based on the prior studies examining the effects of GVS on the H-reflex (Kennedy and Inglis, 2001, 2002; Lowrey and Bent, 2009), GVS was administered at the CTIs of 60, 90, 110, 140, 160, 190, 220, and 250 ms. Note that the duration of the GVS conditioning stimulus was intentionally prolonged in relation to the longest CTI. i.e., 250 ms, to reduce confounding off-stimulation effects (Kennedy and Inglis, 2001). The participants reported that the intensity of the perception of head displacement decreased toward the end of the experimental session, thus, to avoid habituation to GVS, each CTI was presented only 6 times at random order, along with 6 test (control) responses.

### Transcranial Magnetic Stimulation (TMS)

TMS was delivered over the right primary motor cortex with single pulses using a Magstim 2 stimulator (Magstim, Dyfed, United Kingdom). Initially, the point where the lines between the inion and glabella, and the left and right ear tragus met was marked. The double cone coil (110 mm of diameter) was placed parallel and approximately 1 cm lateral to the right from this intersection point (Knikou, 2014). With the coil held at this position and orientated to produce posterior-to-anterior currents, the stimulation intensity was gradually increased until the motor evoked potentials (MEPs) in the quiescent left leg muscles (e.g., VL, RF, MH, TA, SOL, and MG) were revealed. The coil was also moved by few mm in different directions so that the minimal stimulation intensity which produced the responses above 50 μV on the left leg muscles was identified. The coil then was maintained in that position during the experiment. Based on the previous studies (Knikou, 2014; Roy et al., 2014), TMS was administered at the CTIs of 10, 20, 30, 90, 110, and 140 ms. Each CTI was presented 6 times at random order, along with 6 test (control) responses.

### Experimental Procedure

The participants were placed in a supine position, and stayed relaxed during the experiments. The background EMG from the leg muscles was monitored throughout the sessions to ensure the quiescent muscles’ state during the control and conditioning stimulation. In addition, we prompted the participants to keep their head position straight, and as stable as possible to avoid turning it on either side, and ensured the symmetrical position of the limbs.

First, spinal stimulation was administered at intensities ranging from 2 to 100 mA, or the maximum tolerable intensity, using 2 mA increments. A minimum of 3 stimuli were delivered per intensity. Recruitment curves were constructed by plotting the magnitude of spinally evoked motor potentials against increasing stimulation intensity at each stimulation location. To assess the modulatory effects of the conditioning stimulation, test stimuli were identified such that their intensity produced smaller responses corresponding to the initial ascending phases of the recruitment curves across all muscles (Sayenko et al., 2015a). Then, conditioning GVS or TMS stimulation was performed on different days with at least 24 h in between them.

### Data Analysis

Magnitudes of the spinally evoked motor potentials were calculated by measuring the peak-to-peak amplitude, within a time window manually selected for each muscle. The onset of the time window was defined from the overlaid responses based on the earliest inclination from the baseline across all stimulation intensities. The duration of the time window for different muscles varied between 20 and 40 ms, and was kept the same for a given muscle (Sayenko et al., 2015a). Magnitudes of the test and conditioned spinally evoked motor potentials were sorted and averaged, and the degree of modulation at different CTIs was determined by comparison with the test responses.

**FIGURE 1:**
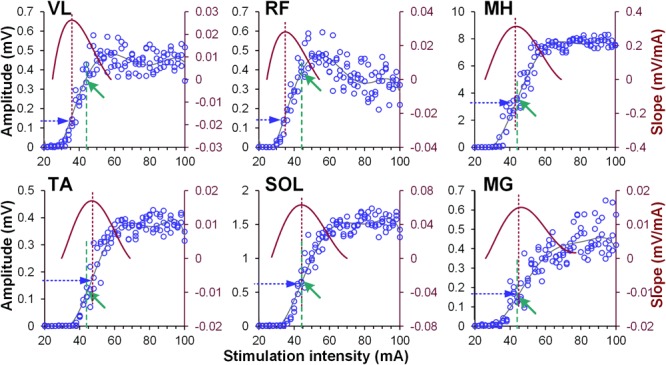
Examples of recruitment curves (blue circles, left Y-axis) of right vastus lateralis (VL), rectus femoris (RF), medial hamstrings (MH), tibialis anterior (TA), soleus (SOL), and medial gastrocnemius (MG) muscles recorded in one participant. A 6th order polynomial function (gray line) was fitted to each recruitment curve. Tangential slope was then computed as the first derivative of the fitted polynomial (brown line, right Y-axis). Maximal tangential slope (RRmax) was calculated from each recruitment curve (vertical brown dashed line). Finally, the response magnitude corresponding to the RRmax was defined (horizontal blue dashed arrow) for normalization of the amount of modulatory effects induced by the conditioning stimulation. Note different magnitude of spinally evoked motor potentials in various muscles (green arrows and vertical green dashed lines), evoked by the single stimulation intensity of 44 mA during conditioning protocol.

It is common that the degree of facilitation is reported as a percentage of the size of the test response. However, as was noted by Crone et al. (Crone et al., 1990) this form of presentation makes it difficult to estimate the “real” amount of excitation reaching the motor neuron pool, especially when comparing conditioning effects obtained across different muscles and participants. Crone et al. (Crone et al., 1990) suggested the absolute increase (e.g., difference between the conditioned and test responses), expressed as a per cent of maximum M-response, as a more direct measure and also a more suitable parameter when interpreting the physiological significance of the changing susceptibility to modulation for test reflexes of different size. However, even though the maximum spinally evoked motor potentials’ amplitude can be revealed in most of the leg muscles of non-injured individuals, in some cases it is difficult to reach a “true plateau” in the magnitude of the responses (Sayenko et al., 2015a) due to either stimulator limitations or tolerance and/or girth of participants. Based on these considerations, the magnitude of modulatory effects induced by the conditioning stimulation was presented as a difference between the conditioned and test responses at each CTI, expressed as a per cent of the response corresponding to the maximal rate of increase in spinally evoked motor potentials magnitude (RRmax) for a given muscle. The rate of increase at each point was given by the tangential slope, which was calculated by fitting a 6th order polynomial function to the recruitment curve and finding the value of its first derivative, using MATLAB (MathWorks, Inc., Natick, MA, United States) (Sayenko et al., 2015a; Figure 1). A 6th order polynomial was chosen empirically because in all cases, the polynomial showed excellent fitting to the ascending limb [root-mean-square error (RMSE) = 166.6 ± 10.0 μV, mean ± standard error of the mean (SEM)], and the point of maximum tangential slope fell within the expected portion of the curve. The conditioning effects for each muscle were submitted to 2 sides (left and right) by 9 or 7 CTIs (for GVS or TMS conditioning, respectively) analysis of variance (ANOVA). *Post hoc* Dunnett’s test was performed to decompose significant effects for each conditioning paradigm (α = 0.05). The results for the pooled data are presented as mean values and standard error of the mean (SEM).

## Results

**FIGURE 2:**
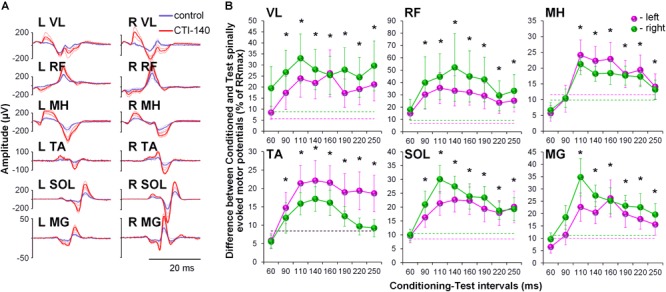
**(A)** Spinally evoked motor potentials obtained for one participant under control conditions (blue traces) and during conditioning via GVS delivered 140 ms prior the spinal stimulus (red traces). The thin traces indicate individual responses, whereas the bold traces indicate the average responses. Shown is the time window between 10 and 40 ms following the stimulus. **(B)** Group average (*n* = 20) showing the conditioning effects of galvanic vestibular stimulation (GVS) delivered at different CTIs on spinally evoked motor potentials in bilaterally recorded leg muscles. The amount of modulation of spinally evoked motor potentials is expressed as a difference between the conditioned and test responses at each CTI, expressed as a per cent of the response corresponding to the maximal tangential slope (RRmax) for a given muscle. Variation of the test (unconditioned) responses in left and right muscles is indicated by pink and green dashed lines, respectively. VL, vastus lateralis; RF, rectus femoris; MH, medial hamstrings; TA, tibialis anterior; SOL, soleus; MG, medial gastrocnemius muscles. The error bars indicate standard error of the mean. Asterisks indicate statistically significant differences between the averaged bilateral control and the conditioned magnitudes of spinally evoked motor potentials (^∗^*p* < 0.05).

No discomfort was reported by the participants during the GVS conditioning procedures. All participants experienced a short-lasting sensation of “dizziness” or “head motion primarily to the right” during cathode-right stimulation. This sensation was perceived as less intensive toward the end of the session, presumably due to habituation to the stimulus. The convergence of the GVS induced volleys on lumbosacral motor pools resulted in pronounced and consistent facilitation of spinally evoked motor potentials, irrespective of the rostro-caudal location of a given motor pool (Figure 2A). Although the tests were performed with the cathode on the right side, the two-way ANOVA revealed no significant side-specific effects in the amount of conditioning of spinally evoked motor potentials in the left and right leg muscles. The effects in some muscles were not consistent across CTIs (e.g., see VL, MH, SOL, and MG) and this may be the reason for the lack of significance of the side-specific effect. Moreover, it appears that in some muscles, there was a higher tendency for the conditioning effects to be larger on the left (TA and MH) and in other muscles – on the right side (VL, RF, SOL, and MG), and this difference may relate roughly to their functional role, that is flexion vs. extension, during movements. The pooled analysis yielded significant effects for the CTIs in all muscles (*F*_8,38_ > 7.0, *p* < 0.01), and of the latencies tested, facilitation was statistically significant at CTIs between 90 and 250 ms, with the greatest magnitude observed at CTIs of 110 and 140 ms (Figure 2B and Supplementary Table 1).

**FIGURE 3:**
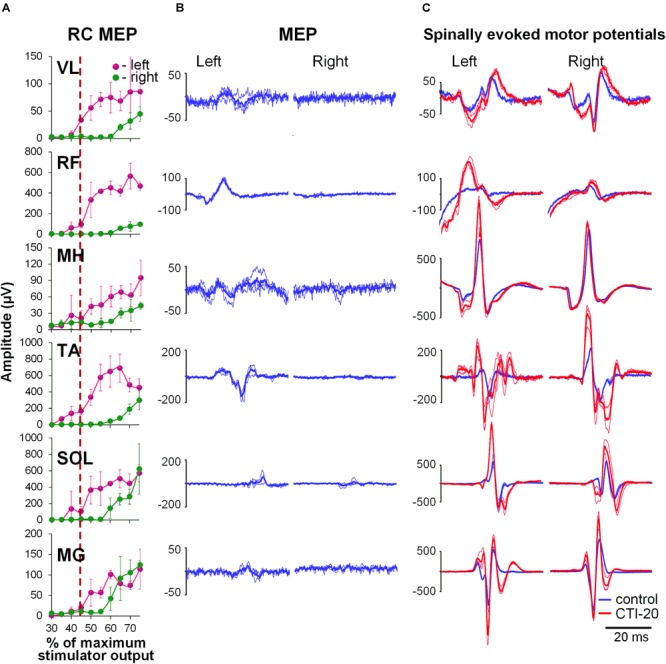
Interaction of spinally evoked motor potentials and MEPs obtained using transcranial magnetic stimulation (TMS) delivered over the right motor cortex for one participant. **(A)** Recruitment curves of MEP in bilaterally recorded leg muscles in one participant. Vertical red dashed line indicates stimulation intensity chosen for conditioning. **(B)** MEPs selected as conditioning stimuli. **(C)** Spinally evoked motor potentials under control conditions (blue traces) and during conditioning via TMS delivered 20 ms prior the spinal stimulus (red traces). The thin traces indicate individual responses, whereas the bold traces indicate the average of 3 responses. Shown are the time windows between 5 and 50 ms (for spinally evoked motor potentials) and between 20 and 65 ms (for MEP) following the stimulus. VL, vastus lateralis; RF, rectus femoris; MH, medial hamstrings; TA, tibialis anterior; SOL, soleus; MG, medial gastrocnemius muscles.

**FIGURE 4:**
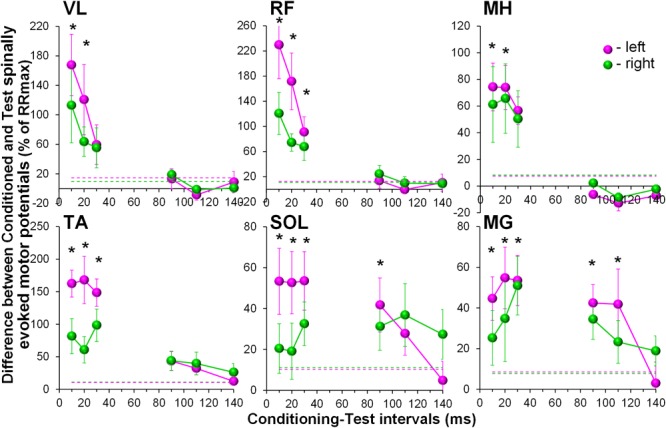
Group average (*n* = 8) showing the conditioning effects of transcranial magnetic stimulation (TMS) delivered over the right motor cortex at different CTIs on spinally evoked motor potentials in bilaterally recorded leg muscles. The amount of modulation of spinally evoked motor potentials is expressed as a difference between the conditioned and test responses at each CTI, presented as a per cent of the response corresponding to the maximal tangential slope (RRmax) for a given muscle. Variation of the test (unconditioned) responses in left and right muscles is indicated by pink and green dashed lines, respectively. VL, vastus lateralis; RF, rectus femoris; MH, medial hamstrings; TA, tibialis anterior; SOL, soleus; MG, medial gastrocnemius muscles. The error bars indicate standard error of the mean. Asterisks indicate statistically significant differences between the averaged bilateral control and the conditioned magnitudes of spinally evoked motor potentials (^∗^*p* < 0.05).

During the conditioning TMS applied over the right motor cortex, cortically mediated descending volleys increased spinal motor output, with greater effects occurring in the contralateral, left leg muscles (Figure 3). Also similar to GVS induced effects, the TMS conditioned responses occurred across all muscles tested. The two-way ANOVA revealed no significant effects in the amount of conditioning between the left and right muscles. In all muscles, the effects were not consistent across CTIs, with a tendency for larger conditioning effects on ipsilateral, left side at shorter 10 to 20 ms CTIs (also 30 ms CTI in RF, TA, and SOL). It is worth noting, that in SOL and MG, the tendency for the left-right difference in the conditioning effects, persisted at longer CTIs, with inconsistent prevalence on either side. Such inconsistency in the side-specificity across shorter and longer CTIs may be the reason for the lack of significance of the main side-specific effects (Figure 4 and Supplementary Table 2). The effects of CTIs were strong, with significant facilitation occurring at considerably shorter CTIs – between 10 and 30 ms (*F*_6,14_ > 3.7, *p* < 0.01). The greatest magnitude occurred at the 10 ms CTI in VL and RF, and 20–30 ms CTI in MH, TA, SOL, and MG. In addition, significant conditioning effects were also revealed at longer CTIs of 90 ms in SOL and MG.

**FIGURE 5:**
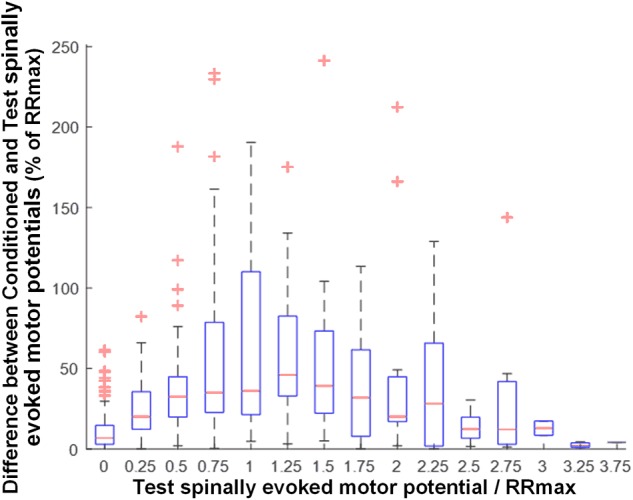
Group data (*n* = 20) illustrating the magnitude of GVS conditioning as a function of unconditioned amplitude of spinally evoked motor potentials, both normalized to RRmax. Bin widths for stimulation intensity are 0.25 of RRmax. The results are summarized in boxplots with the box as the 25–75th percentile, and the whiskers as the highest and lowest values. Red lines inside the boxes indicate the mean values. Crosses represent outlying conditioning responses. Value 1 denotes RRmax.

Activation threshold of spinally evoked motor potentials in different muscles occurred at different stimulation intensities, due to the fact that a stimulation delivered at a single location activates the extent of motor pools along the rostro-caudal axis of the lumbosacral enlargement (cf. Figure 1). As such, at a given intensity, the evoked potentials were of various amplitudes (relative to maximum), across proximal and distal muscles. The common feature of the conditioning effects in all muscles obtained each at a different point of the recruitment curves, was, however, that the greatest magnitude of conditioning always occurred at or near the maximum rate of recruitment (RRmax) (Figure 5).

## Discussion

The convergence of descending volleys elicited by conditioning stimulation using GVS or TMS, on lumbosacral motor pools results in excitatory neuromodulation of spinally evoked motor potentials in lower limb muscles bilaterally. The difference in temporal manifestation of the observed phenomena indicates different routes of transmission for each form of descending volley. Our work demonstrates the utility and sensitivity of transcutaneous spinal cord stimulation in human neurophysiological studies.

### Spinally Evoked Motor Potentials

Previous electrophysiological (Maertens de Noordhout et al., 1988; Hunter and Ashby, 1994; Minassian et al., 2007a) and computational (Rattay et al., 2000; Ladenbauer et al., 2010; Danner et al., 2011) studies demonstrated that the structures, stimulated electrically by epidural or transcutaneous spinal lumbar spinal cord stimulation, are primarily large-to-medium diameter proprioceptive and cutaneous afferents within lumbar and upper sacral posterior rootlets/roots. The prevailing view is that, the motor effects of lumbar spinal stimulation are initiated through the recruitment of posterior-root fibers, rather than through the direct electrical stimulation of spinal gray matter neurons, and depend on the applied stimulation parameters, such as location, intensity, and frequency (Dimitrijevic et al., 1986; Jilge et al., 2004; Sayenko et al., 2014; Gerasimenko et al., 2015; Hofstoetter et al., 2015b; Rejc et al., 2015; Minassian et al., 2016; Grahn et al., 2017). The induced afferent input transsynaptically projects to both homonymous and heteronymous motor pools (Murg et al., 2000; Minassian et al., 2016), as well as interneurons, primarily in the intermediate laminae (Minassian et al., 2007b; Danner et al., 2015; Gerasimenko et al., 2015, 2018; Hofstoetter et al., 2015a; Sayenko et al., 2018). Single pulses delivered with lower stimulation intensities result in initial preferential recruitment of lower threshold afferent fibers accompanied to some extent with involvement of motor axons, whereas, with increasing intensity, more motor axons become activated, leading to the decreased latency of the response and causing an occlusion effect of the afferent pathways (Roy et al., 2012; Sayenko et al., 2015a). In the present experiments, the spinal stimulation location and intensity were adjusted such that activation thresholds occurred at lower stimulation intensities possible, and the magnitude of responses in all muscles was submaximal. This minimized the possibility of direct motor axons stimulation, which would prevent conditioning modulation of spinally evoked motor potentials. The fact that the potentials were conditioned indicates presynaptic excitation of posterior-root fibers induced by spinal stimulation. Examining the properties of individual spinally evoked motor potentials when paired pulses are applied at interstimuli intervals of 30–50 ms in future experiments could help in dissecting the role of afferent vs. efferent routs of activation during testing spinal stimulation (Roy et al., 2012; Sayenko et al., 2015b; Danner et al., 2016; Minassian et al., 2016).

### Conditioning Effects of GVS

Vestibular stimulation enhanced the magnitude of the spinally evoked motor potentials in each of the leg muscles, reflecting changes in the excitability of the projecting lumbosacral motor pools. One of our initial hypotheses included a premise that the conditioning effects of GVS delivered through the cathode placed on the right side, would depend on the rostro-caudal or mediolateral motor pool location within the lumbosacral enlargement, or their functional role in motor control (Shapovalov et al., 1966; Kostyuk, 1967; Pierrot-Deseilligny and Burke, 2005). This hypothesis was not supported. One of the most plausible explanations for the generalized conditioning effects can be attributed to a resting, supine position of the participants during the experiment. Until recently, it was unclear whether it is possible to elicit a response in muscles that are not currently engaged in the maintenance of balance. Modulation of EMG responses in human lower limb muscles during GVS has been well documented in human subjects during standing (Iles and Pisini, 1992b; Fitzpatrick and Day, 2004). On the contrary, results from experiments performed using the soleus H-reflex with the subject is in sitting or lying positions are incongruent and vary from marginal modulation (Kennedy and Inglis, 2001, 2002; Ghanim et al., 2009) to strong facilitation (Lowrey and Bent, 2009). Our data are novel in that it demonstrates substantial excitatory responses from the multiple quiescent leg muscles. These features, in turn, allow standardization of the experimental condition, and make it feasible to utilize this experimental paradigm in clinical populations, when voluntary contraction or actively maintained postures are hard or impossible to achieve.

Can the generalized conditioning effects revealed in both left and right lower limb motor pools be due to the summation of simultaneous vestibular and cutaneous afferent inputs at the spinal level through vestibulospinal and reticulospinal tracts?

It has been demonstrated in previous experiments, that an electrical stimulus delivered over the mastoid processes during GVS may also excite local cutaneous afferents in the skin, and produce a startle response, further contributing to the reticulospinal volley (Ghanim et al., 2009; Lowrey and Bent, 2009). However, in our experimental setup we ensured that GVS did not produce any uncomfortable sensations, such as pinching, and the used GVS intensity was well tolerated. As such, the observed lack of muscle- and side-specific conditioning effects rather support the notion that GVS induced responses are not conveyed exclusively through the vestibulospinal pathways and can be transmitted via reticulospinal tracts, as suggested previously (Pierrot-Deseilligny and Burke, 2005; Lowrey and Bent, 2009). Finally, previous studies investigating the conditioning effects of vestibular (Kennedy and Inglis, 2001, 2002; Ghanim et al., 2009; Lowrey and Bent, 2009), auditory (Delwaide and Schepens, 1995; Ilic et al., 2011), and arm afferent (Meinck and Piesiur-Strehlow, 1981; Kagamihara et al., 2003) stimulation on the soleus H-reflex modulation, also demonstrated peak facilitatory effects at CTI of approximately 100 to 120 ms. This striking similarity in the temporal facilitation, regardless of the conditioning stimulation location, may be attributed to a common integrative mechanism at a pre-motor neuronal level. Although the evidence for such an integrative system, referred to as common ‘lumbar propriospinal neurones’ is available from experimental animal models (Pierrot-Deseilligny and Burke, 2005), it has not yet been directly demonstrated in human. This propriospinal system is organized advantageously to integrate supraspinal, spinal, and sensory signals to ensure motor neuron pools receive the most recent and accurate information and commands (Flynn et al., 2011). Evidence indicates that short-axon lumbar propriospinal neurons transmit the descending commands to lower-limb motor neurons, and receive various amount of convergence from descending pathways (Kostyuk, 1967; Shapovalov, 1975; Schomburg, 1990; Pierrot-Deseilligny and Burke, 2005). Our data, although indirectly, support the existence of such pre-motor neuronal integrative system in human. It is worth noting that the location, projection pattern and dynamic role of the propriospinal system, provides this subset of interneurons a particularly critical role in neuroplasticity following SCI (Flynn et al., 2011).

Regardless of the specific underlying mechanisms, it appears that a change in the vestibular afferent firing rate via GVS affects multisegmental interneurons that regulate motoneuron excitability. However, in contrast to TMS induced modulation, which prominent facilitatory effects are consistently observed at much shorter latencies, GVS induced modulation always occurred at longer latencies. Although TMS can evoke longer latency modulation of spinally evoked motor potentials in distal muscles, this can be distinguished from the long-lasting temporal manifestation of the GVS induced conditioning effects in all muscles tested. These distinctions provide opportunity for quantitative assessment and comparison of residual supraspinal connectivity to lumbosacral sensorimotor networks following CNS injury/disease, as well as examination of polysynaptic vs. monosynaptic corticospinal connectivity. Given recent studies implicating specific pathways in mediating adaptive plasticity resulting in the re-establishment of descending input to sublesional networks in multiple mammalian models of SCI including non-human primates (Capogrosso et al., 2016; Asboth et al., 2018), discrimination between these anatomically and functionally distinct pathways holds potential as biomarkers for potential recovery after human SCI.

### Conditioning Effects of Transcranial Magnetic Stimulation (TMS)

Our observations of the spatiotemporal convergence between cortical and spinal cord stimulation were expected and agree with the previous reports that corticospinal excitation may produce a net depolarization of motor neurons, thereby converging with spinal cord stimulation (Knikou, 2014; Roy et al., 2014; Andrews et al., 2015).

Short-latency responses occurring predominantly on the left side strongly supports that the onset of the excitation involves monosynaptic corticospinal transmission (Valls-Sole et al., 1994; Serranova et al., 2008; Costa et al., 2011). Some conditioning effects on the ipsilateral, right side, most likely are attributed to relatively high TMS intensity required to produce supra-threshold MEPs in left leg quiescent muscles, and thus, unintentionally involving the right motor cortex.

Interestingly, the long-latency effects revealed in our study were most pronounced at the 90 and 110 ms CTIs in plantarflexor muscles only. It is unclear why that was the case. It is known that the strength of the projections of individual spinal pathways on different motoneuron pools have undergone phylogenetic adaptations to different motor repertoires in human and animals (Pierrot-Deseilligny and Burke, 2005). In human lower limbs, more elaborate reflex organization in plantarflexors, including their descending control, is required for bipedal stance and gait, and, as such, can be attributed to the revealed effects. In addition, common propriospinal interneurons indicated above may contribute to the long-latency TMS conditioned effects. Indeed, it was demonstrated in experimental animal models that the ventromedial propriospinal neurons located in L3-L5 segments, transmitting the descending commands to distal muscles, predominantly receive inputs from corticospinal tract (Kozhanov and Shapovalov, 1977; Pierrot-Deseilligny and Burke, 2005).

TMS has been used to assess the function of the corticospinal tract and its integrity (Dimitrijevic et al., 1992; McKay et al., 1997, 2005). However, during certain neurological conditions, such as motor complete SCI, MEPs in leg muscles are often not detected in response to TMS, even during a voluntary muscle contraction attempt (McKay et al., 2005; Angeli et al., 2014), which precludes a quantitative evaluation of translesional corticospinal connectivity. The substantial excitatory effects observed in the quiescent leg muscles using TMS conditioning in our study suggests that this approach might bring increased sensitivity to the evaluation of corticospinal tract function after SCI.

### Magnitude of Modulation of Spinally Evoked Motor Potentials Is Dependent Upon Rate of Recruitment

Our results demonstrate the greatest magnitude of modulation of spinally evoked motor potentials occurred when the test response was at or near RRmax (Figure 5). The maximal slope of the recruitment curve in different muscles has been suggested to indicate the rate of afferent recruitment (Funase et al., 1994; Komiyama et al., 1999; Sekiguchi et al., 2003), may depend on the tonic level of presynaptic inhibition (Pierrot-Deseilligny and Burke, 2005; Sayenko et al., 2015a), and may be attributed to the type, number, and size of the motor neurons (Pierrot-Deseilligny and Burke, 2005). Thus, the utilized normalization approach is supported by the physiological meaning of RRmax, and can serve as a real-time or *post hoc* technique during neurophysiological or functional assessment, when a quantitative characterization of the conditioning effects among different motor pools is advantageous.

### Limitations

While there are several advantages in the use of TSS to monitor motor neuron excitability in electrophysiological studies, some limitations remain. Direct comparison of the GVS and TMS induced effects is difficult due to the difference in intensity and duration of the conditioning stimuli. As our methods were designed to be easily transferred to a clinical population with limited mobility, a supine testing position was ideal. However, this limits our ability to interpret the findings on postural and motor control.

## Conclusion

Transcutaneous spinal stimulation, incorporated into previously identified methods for evaluating pathway-specific convergence of afferent and descending influences on spinal motor output, allows observation of modulatory effects at spinal network level. Characterization of excitatory short- and long-latency conditioning effects in multiple lumbosacral motor pools bilaterally, obtained through anatomically and functionally distinctive descending pathways, demonstrates the diagnostic utility of transcutaneous electrical spinal cord stimulation in human neurophysiological studies. The complexity of the GVS-induced conditioning effects on spinally evoked motor potentials is consistent with the vestibulospinal pathways anatomically and functionally interfacing with other descending systems, including the reticulospinal pathway. The difference in the pattern of temporal-dependent modulation of supraspinal-spinal connectivity in response to GVS and TMS, may allow differentiation of particular pathway in the re-establishment of voluntary control after complete paralysis. By investigating spinally evoked motor potentials recorded bilaterally from proximal and distal lower limb muscles, we show the feasibility of a comprehensive assessment of the specificity as well as magnitude of the existing or *de novo* connectivity to the lumbosacral motor pools. The characterization of the quality of supraspinal-spinal network can guide more injury-specific individuality in tailoring activity-dependent treatments to improve motor function.

## Author Contributions

DS, DA, VE, SH, and YG designed the study. DS, DA, AM, and KG collected the data. DS, DA, AM, KG, and SH developed the methodological aspects of analysis and data interpretation. DS prepared the figures and drafted the manuscript. All authors revised the manuscript, approved the final version, and agree to be accountable for all aspects of the work in ensuring that questions related to the accuracy or integrity of any part of the work are appropriately investigated and resolved.

## Conflict of Interest Statement

YG and VE hold shareholder interest in NeuroRecovery Technologies and hold certain inventorship rights on intellectual property licensed by The Regents of the University of California to NeuroRecovery Technologies and its subsidiaries. The remaining authors declare that the research was conducted in the absence of any commercial or financial relationships that could be construed as a potential conflict of interest.
