# Supplementary material for: Vestibulospinal and Corticospinal Modulation of Lumbosacral Network Excitability in Human Subjects
Source: Front Physiol. 2018 Dec 6;9:1746. doi: 10.3389/fphys.2018.01746 (PMC6291495; doi:10.3389/fphys.2018.01746)
Supplement: Supplementary file 1 [file Table_1.DOCX]

**Supplementary Table 1.** Analysis of Variance Results of GVS conditioning effects.

***m. Vastus lateralis (VL)***

| Source | DF | SS | MS | F | P |
| --- | --- | --- | --- | --- | --- |
| Total | 359 | 497278.6 | 1385.177 |  |  |
| Side | 1 | 3891.303 | 3891.303 | 0.419142 | 0.52126 |
| CTI | 8 | 24171.6 | 3021.451 | 7.995555 | < .0001 |
| Interaction | 8 | 1545.421 | 193.1776 | 0.511199 | 0.84778 |
| Subjects (matching) | 38 | 352791.3 | 9283.981 | 24.56786 | < .0001 |
| Error | 304 | 114879 | 377.8913 |  |  |

***m. Rectus femoris (RF)***

| Source | DF | SS | MS | F | P |
| --- | --- | --- | --- | --- | --- |
| Total | 359 | 1415705.9 | 3943.4705 |  |  |
| Side | 1 | 7273.8777 | 7273.8777 | 0.2700717 | 0.6063 |
| CTI | 8 | 59490.771 | 7436.3464 | 7.0020369 | < .0001 |
| Interaction | 8 | 2626.1519 | 328.26899 | 0.30909689 | 0.96233 |
| Subjects (matching) | 38 | 1023459.1 | 26933.135 | 25.360143 | < .0001 |
| Error | 304 | 322855.96 | 1062.0262 |  |  |

***m. Medial hamstring (MH)***

| Source | DF | SS | MS | F | P |
| --- | --- | --- | --- | --- | --- |
| Total | 359 | 105468.9 | 293.7852 |  |  |
| Side | 1 | 210.88 | 210.88 | 0.163457 | 0.68826 |
| CTI | 8 | 18431.19 | 2303.898 | 18.67728 | < .0001 |
| Interaction | 8 | 302.6756 | 37.83446 | 0.306717 | 0.96321 |
| Subjects (matching) | 38 | 49024.85 | 1290.128 | 10.45883 | < .0001 |
| Error | 304 | 37499.3 | 123.353 |  |  |

***m. Tibialis anterior (TA)***

| Source | DF | SS | MS | F | P |
| --- | --- | --- | --- | --- | --- |
| Total | 359 | 110928.7 | 308.9936 |  |  |
| Side | 1 | 2272.694 | 2272.694 | 1.427693 | 0.23955 |
| CTI | 8 | 14773.15 | 1846.644 | 17.31564 | < .0001 |
| Interaction | 8 | 971.5893 | 121.4487 | 1.138802 | 0.33687 |
| Subjects (matching) | 38 | 60490.87 | 1591.865 | 14.92663 | < .0001 |
| Error | 304 | 32420.39 | 106.646 |  |  |

***m. Soleus (SOL)***

| Source | DF | SS | MS | F | P |
| --- | --- | --- | --- | --- | --- |
| Total | 359 | 131300.4 | 365.73928 |  |  |
| Side | 1 | 653.81413 | 653.81413 | 0.38547906 | 0.53839 |
| CTI | 8 | 21638.312 | 2704.789 | 18.777263 | < .0001 |
| Interaction | 8 | 766.19708 | 95.774636 | 0.6648894 | 0.72235 |
| Subjects (matching) | 38 | 64452.104 | 1696.108 | 11.774769 | < .0001 |
| Error | 304 | 43789.974 | 144.04597 |  |  |

***m. Medial gastrocnemius (MG)***

| Source | DF | SS | MS | F | P |
| --- | --- | --- | --- | --- | --- |
| Total | 359 | 183071.49 | 509.94845 |  |  |
| Side | 1 | 1889.5399 | 1889.5399 | 0.90961133 | 0.34625 |
| CTI | 8 | 27061.582 | 3382.6977 | 13.915837 | < .0001 |
| Interaction | 8 | 1285.6826 | 160.71032 | 0.66113461 | 0.72562 |
| Subjects (matching) | 38 | 78937.579 | 2077.3047 | 8.5456743 | < .0001 |
| Error | 304 | 73897.11 | 243.0826 |  |  |

DF = degrees of freedom; SS = sum of squares; MS = mean sum of squares; F = F ratio; P = P value.
