# Supplementary material for: Vestibulospinal and Corticospinal Modulation of Lumbosacral Network Excitability in Human Subjects
Source: Front Physiol. 2018 Dec 6;9:1746. doi: 10.3389/fphys.2018.01746 (PMC6291495; doi:10.3389/fphys.2018.01746)
Supplement: Supplementary file 2 [file Table_2.DOCX]

**Supplementary Table 2.** Analysis of Variance Results of TMS conditioning effects.

***m. Vastus lateralis (VL)***

| Source | DF | SS | MS | F | P |
| --- | --- | --- | --- | --- | --- |
| Total | 111.00 | 824339.19 | 7426.48 |  |  |
| Side | 1 | 7063.984 | 7063.984 | 0.709946 | 0.41363 |
| CTI | 6 | 294599.8 | 49099.96 | 11.3057 | < .0001 |
| Interaction | 6 | 18568.05 | 3094.675 | 0.712576 | 0.64042 |
| Subjects (matching) | 14 | 139300.4 | 9950.026 | 2.291081 | 0.01036 |
| Error | 84 | 364807 | 4342.941 |  |  |

***m. Rectus femoris (RF)***

| Source | DF | SS | MS | F | P |
| --- | --- | --- | --- | --- | --- |
| Total | 111.00 | 991165.70 | 8929.42 |  |  |
| Side | 1 | 25226.8 | 25226.8 | 3.02236 | 0.10406 |
| CTI | 6 | 455323 | 75887.16 | 19.28358 | < .0001 |
| Interaction | 6 | 63194.42 | 10532.4 | 2.676374 | 0.01997 |
| Subjects (matching) | 14 | 116854.1 | 8346.722 | 2.120973 | 0.0183 |
| Error | 84 | 330567.4 | 3935.326 |  |  |

***m. Medial hamstring (MH)***

| Source | DF | SS | MS | F | P |
| --- | --- | --- | --- | --- | --- |
| Total | 111.00 | 284469.19 | 2562.79 |  |  |
| Side | 1 | 59.24162 | 59.24162 | 0.016705 | 0.899 |
| CTI | 6 | 130699.9 | 21783.31 | 17.8434 | < .0001 |
| Interaction | 6 | 1513.107 | 252.1845 | 0.206572 | 0.97385 |
| Subjects (matching) | 14 | 49649.31 | 3546.379 | 2.904951 | 0.00127 |
| Error | 84 | 102547.7 | 1220.805 |  |  |

***m. Tibialis anterior (TA)***

| Source | DF | SS | MS | F | P |
| --- | --- | --- | --- | --- | --- |
| Total | 111 | 630135.3 | 5676.895 |  |  |
| Side | 1 | 26767.24 | 26767.24 | 3.629703 | 0.07751 |
| CTI | 6 | 267527.8 | 44587.97 | 21.25465 | < .0001 |
| Interaction | 6 | 56382.21 | 9397.035 | 4.479475 | 0.00055 |
| Subjects (matching) | 14 | 103243 | 7374.499 | 3.515352 | 0.00016 |
| Error | 84 | 176215.1 | 2097.799 |  |  |

***m. Soleus (SOL)***

| Source | DF | SS | MS | F | P |
| --- | --- | --- | --- | --- | --- |
| Total | 111.00 | 150079.11 | 1352.06 |  |  |
| Side | 1 | 2518.413 | 2518.413 | 1.132158 | 0.30532 |
| CTI | 6 | 22084.93 | 3680.821 | 3.705495 | 0.00257 |
| Interaction | 6 | 10893.02 | 1815.504 | 1.827674 | 0.10339 |
| Subjects (matching) | 14 | 31142.1 | 2224.436 | 2.239347 | 0.01233 |
| Error | 84 | 83440.65 | 993.3411 |  |  |

***m. Medial gastrocnemius (MG)***

| Source | DF | SS | MS | F | P |
| --- | --- | --- | --- | --- | --- |
| Total | 111.00 | 155844.87 | 1404.01 |  |  |
| Side | 1 | 1578.082 | 1578.082 | 0.502397 | 0.49008 |
| CTI | 6 | 33240.19 | 5540.031 | 6.386705 | < .0001 |
| Interaction | 6 | 4186.859 | 697.8098 | 0.804455 | 0.56925 |
| Subjects (matching) | 14 | 43975.47 | 3141.105 | 3.621155 | 0.00011 |
| Error | 84 | 72864.27 | 867.4318 |  |  |

DF = degrees of freedom; SS = sum of squares; MS = mean sum of squares; F = F ratio; P = P value.
